# Ultrahigh UV Responsivity Quasi-Two-Dimensional Bi_x_Sn_1−x_O_2_ Films Achieved through Surface Reaction

**DOI:** 10.3390/ma16216988

**Published:** 2023-10-31

**Authors:** Zhihao Xu, Miao Xu, Fang Chen, Rui Zhai, You Wu, Zhuan Zhao, Shusheng Pan

**Affiliations:** 1 School of Physics and Materials Science, Guangzhou University, Guangzhou 510006, China; 18198435928@163.com (Z.X.); 2112019071@e.gzhu.edu.cn (M.X.); cf.chenfang@e.gzhu.edu.cn (F.C.); rui15561862917@163.com (R.Z.); 2112119088@e.gzhu.edu.cn (Y.W.); 2 Research Center for Advanced Information Materials (CAIM), Huangpu Research and Graduate School of Guangzhou University, Guangzhou 510006, China; 3 Songshan Lake Materials Laboratory, Dongguan 523808, China; 4 Key Lab of Si-Based Information Materials & Devices and Integrated Circuits Design, Department of Education of Guangdong Province, Guangzhou 510006, China

**Keywords:** Bi-doped SnO_2_ (BTO), quasi-two-dimensional materials, responsivity, surface reaction

## Abstract

In this study, quasi-two-dimensional Bi_x_Sn_1−x_O_2_ (BTO) thin films were fabricated using a liquid metal transfer method. The ultraviolet (UV) photodetector based on BTO thin films was constructed, and the ultrahigh responsivity of 589 A/W was observed at 300 nm UV light illumination. Interestingly, by dropping ethanol during light-off period, the recovery time induced by the persistent photoconductivity (PPC) effect is reduced from 1.65 × 10^3^ s to 5.71 s. Furthermore, the recovery time can also be reduced by dropping methanol, propylene glycol, NaNO_2_, and Na_2_SO_3_ after light termination. The working mechanisms are attributed to the rapid consumption of holes stored in BTO thin films by reaction with those solutions. This work demonstrates that the BTO thin films have potential applications in high-performance UV detectors and present an innovation route to weaken the PPC effects in semiconductors by introducing chemical liquids on their surface.

## 1. Introduction

Over the past decades, metal oxide semiconductors have attracted considerable interest in various applications, such as transparent thin-film transistors, [1,2] transparent conduction electrodes, [3] and optoelectronics [4,5]. Among the optoelectronic devices, ultraviolet (UV) photodetectors (PDs) are of particular interest due to their wide applications in plume detection, optical communications, and biomedical sensing [6,7,8,9,10,11]. UV photodetectors fall into many types, including photomultiplication (PM) type, photovoltaic type, and photoconductive type [12]. Photoconductive PDs are among the most attractive candidates due to their high responsivity, simple fabrication process, and durable performance [13,14]. The high responsivity and high internal photoconductive gain are primarily attributed to photocarriers being captured by defects within the material. However, one notable drawback associated with photoconductive PDs is the phenomenon known as the persistent photoconductivity (PPC) effect [15,16]. PPC effect refers to the photoconductivity that can persist for a long time (>1 s) after the terminating light source, which prevents the rapid recovery of the initial undisturbed state. The PPC effects prolong the response times of UV photoconductors and hinder the rapid recovery of the performance of UV photodetectors [17]. Therefore, reducing the PPC effect is an urgent need in photoconductive PDs.

To address this issue, many approaches have been proposed, such as introducing light pulses [18] and electric fields [19,20] in three-terminal devices, surface assembly of organic molecules [21], infrared irradiation [22], and heating [23,24]. For example, Sanghun et al. [18] iminate the PPC effect effectively by applying the pulsed voltage in the three-terminal device, but this device contains a stack of GIZO (25 nm)/IZO (50 nm)/GIZO (17 nm) layers and incorporates an inverted staggered switch-TFT and photo-TFT, which is challenging to fabricate. Pinto et al. [25] reported a phenyl-C_61_-butyric acid methyl ester (PCBM)/rubrene crystal bilayer structure with enhanced sensitivity of 4 × 10^4^ s and a maximum photoresponsivity of 20 A/W, but the decay time in their time-resolved photosensitivity test is still in the range of a few seconds. Sun et al. [24] revealed that continuously pulsed heating can reduce the decay time of a suspended AlGaN/GaN heterostructure photodetector by 30–45% compared to the DC heating mode. However, in most cases, the decay time is just reduced from days or several hours to hundreds of seconds. There is no method that can suppress the PPC effect completely. Furthermore, the use of the aforementioned methods present challenges and is still under investigation due to the requirement of high electric fields, extra facilities, and complicated process [20,22]. The development of cheap and simple methods, along with the identification of suitable materials to eliminate the PPC effect is urgently needed.

SnO_2_ is a promising candidate for UV detection due to its outstanding optical and electrical properties [26,27,28]. The SnO_2_-based photodetectors with photoconductive mode usually exhibit ultrahigh responsivity (>10^3^ A/W) in the UV light range. Unfortunately, the recovery process of the corresponding photodetectors is relatively slow [29,30,31] due to the PPC effect. Some efforts have been implemented to improve device performance by achieving better crystal quality and constructing low-dimension SnO_2_ nanostructures [4,32,33]. For example, Liu et al. [4] prepared a single SnO_2_ microrod photoconductor and demonstrated it has high photoconductive gain (≈1.5 × 10^9^) and quick recovery speed (<1 s). The PPC effect in this work is eliminated by bending and straightening the microrod and subsequently applying a voltage pulse. Nevertheless, achieving such high-performance characteristics requires precise control of the radius of curvature of the bent film and the application of appropriate voltages. These requirements still present challenges in terms of complexity and cost. As an alternative, barium titanate (BTO) has attracted widespread interest in making electronic devices in recent years, including microwave electronics, actuators, piezoelectric transducers, and infrared detectors owing to its large dielectric constant, ferroelectricity, and piezoelectric characteristics [34,35,36,37]. In our previous study, a deep UV photodetector based on Bi_x_Sn_1−x_O_2_ (2D-BTO) (0.017 < x < 0.041) was fabricated and it exhibits a dark current as low as 0.25 nA and a UV responsivity as high as 60 A/W [38]. The recovery speed can be reduced to approximately 1 s if this photodetector is exposed to an ethanol stream environment. This study offers a new possibility to eliminate the PPC effect by exposing the photodetectors to special chemical liquids.

In this study, we have fabricated quasi-two-dimensional Bi_x_Sn_1−x_O_2_ (2D-BTO) thin films (x < 0.002) using the liquid metal transfer method. The photodetector based on 2D-BTO exhibits a high responsivity of 589 A/W and detectivity of 6.82 × 10^12^ Jones at 300 nm. The recovery speed of the resultant photoconductor is dramatically enhanced by introducing various reductive steam/drops on the device surface during the light-off period. The PPC effect is dramatically weakened due to consuming the photogenerated carriers with introduced chemicals, such as ethanol, propylene glycol, NaNO_2_, and Na_2_SO_3_.

## 2. Experimental Details

### 2.1. Sample Preparation

**Figure 1:**
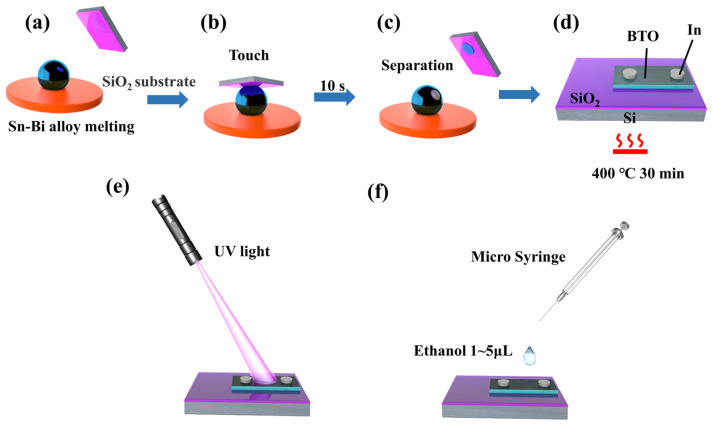
Schematic diagram of quasi-2D-BTO films fabrication process and UV irradiation (**a**) heat melted Sn-Bi alloy, (**b**) silicon oxide substrate in contact with liquid metal, (**c**) separation of silicon oxide substrates from liquid metal, (**d**) Bi-doped SnO_X_ film was annealed at 400 °C for 30 min, (**e**) photoelectric performance test of BTO film, (**f**) ethanol was added to the BTO film after irradiation.

The fabrication process of the BTO films was conducted in a class 1000 clean room (see Figure 1). First, 10 g Sn is heated and melted in the heating stage at a temperature of 280 °C. Then, 1 g of Bi was added to the Sn to form a Bi-Sn alloy (Figure 1a) at a temperature of 280 °C. The formed rough oxide layer generated on the melted Bi-Sn surface was removed using a smooth glass. The smooth glass is inserted into a height-adjustable workbench and then moved in a direction parallel to the Bi-Sn alloy by applying a pushing force. Under the action of the thrust, the rough oxide layer is removed. When the rough oxide layer was removed, this sample was exposed to air for 20 s to obtain a more uniform oxide layer through a non-self-limiting oxidation reaction. Subsequently, a preheated substrate was used to touch the liquid metal for 10 s (Figure 1b). The used substrate is a p-type doped (100) crystal-oriented wafer with a SiO_2_ top layer of 300 nm thickness. A tweezer was used to carefully lift the substrate from liquid metals in the perpendicular direction. Since the melted Bi-Sn alloy has a lower surface energy than the SiO_2_ substrate, it tends to wet and adhere to the solid surface. In addition, due to van der Waals forces, the atoms and molecules on the contact area are attracted, which further contributes to the adhesion between the liquid metal and the substrate surface. Thus, a Bi-doped SnO_x_ film was transferred to the substrate. When the substrate and the liquid metal were separated, a Bi-doped SnO_x_ film was transferred to the substrate under van der Waals forces (Figure 1c), followed by an annealing process at 400 °C for 30 min to obtain BTO films (Figure 1d). Finally, two electrodes were mounted on the BTO thin films by an e-beams evaporation machine (TEMD 500, Beijing Technol Science Co., Ltd., Beijing, China) through a self-designed mask that retains two holes to allow the evaporated material to pass through. The used e-beam power is 20 KW, the energy is 5 MeV, and the current is 4 MA. The fabricated BTO thin films were irradiated under 5 V-biased 365 nm UV light (Figure 1e) for 20 s ~ 5 min. After the termination of the light source, 0.05 mL ethanol was dropped into the sample surface through a plastic injector (Figure 1f).

### 2.2. Measurement and Characterization

Keysight B1500A semiconductor device parameter analyzer was utilized to test the photocurrent and responsivity of fabricated BTO films. The used voltage is 5 V, and the illumination wavelength of UV light is 365 nm. The X-ray Photoelectron Spectroscopy (XPS, Nexsa, Thermo scientific, Waltham, MA, USA) was used to measure the element valences. The XPS uses an Ag Lα excitation source. The full spectrum, O, Sn, and Bi spectra were extracted, respectively. An atomic force microscope (AFM, dimension icon, Bruker Corporation, Billerica, MA, USA) was used to test the thickness of the prepared BTO thin films. The sample surface was scanned by AFM, and the height variations across the whole surface were collected. The average thickness is calculated using collected topographic data from the AFM scan. Absorption spectra were performed using a UV-VIS spectrophotometer (UV-Vis, UV-3600i PULS, Shimadz Corporation, Kyoto, Japan). The absorption spectra were measured from 200 to 1000 nm. Surface micromorphology was observed by a Metallographic microscope (Meta test E1-M, Metatest Corporation, Nanjing, China) and a Transmission Electron Microscopy (JEM-F2000, Jeol, Tokyo Metropolis, Japan). The used electron beam acceleration voltage is 100 kV, and the magnification is 5000× and 50,000×. The samples prepared for TEM observation were cut by a focused ion beam machine (ZEISS-AURIGA, Carl Zeiss AG, Oberkochen, Germany).

## 3. Results and Discussion

### Structure and Elements

**Figure 2:**
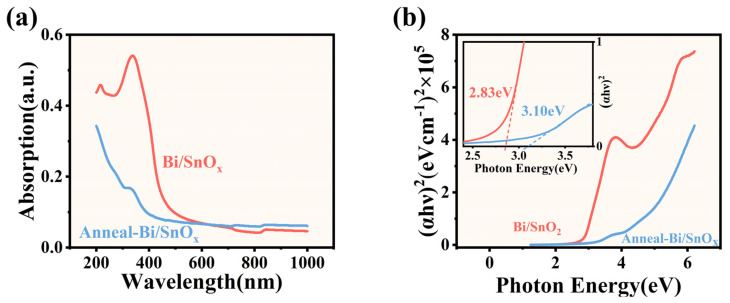
(**a**) The absorption spectra of BTO thin film before and after thermal annealing at 400 °C, (**b**) the ${\left(\alpha hv\right)}^{2}-hv$ of BTO thin film before and after thermal annealing at 400 °C.

Figure 2a shows the optical absorption spectra of 2D-BTO films before and after annealing at 400 °C for 30 min in an oxygen atmosphere. After thermal treatment, the absorption edge shifts towards the lower wavelength side, which indicates the optical band gap of 2D-BTO films becomes wider. And the absorbance is reduced after annealing treatment. This is because the BTO film becomes more transparent after annealing, which involves the partial conversion of SnO to SnO_2_ after thermal treatment in an oxygen atmosphere [39]. In addition, Bi doping can lead to changes in the bandgap energy of SnO2 when annealing, which reduces the absorbance of the prepared BTO thin films. The ${\left(\alpha hv\right)}^{2}$ was depicted in Figure 2b. The optical band gap of 2.78 eV of as-prepared BTO films was obtained by linearly extrapolating towards zero absorption. The band gap is consistent with the reported values of SnO [40], which further illustrates that dominant oxides are SnO in the as-prepared BTO films. After annealing, the optical band gap increases to 3.10 eV, which is close to the reported values of SnO_2_ films [41]. The optical band gap increasing after the annealing of BTO films can be partially attributed to the crystal structure changes, since the annealing process causes the atoms in the BTO film to rearrange, forming a more ordered and perfect crystal lattice. This increased order causes changes in the material’s electronic structure, affecting its band gap. On the other hand, Bi doping and annealing can help reduce defects and vacancies in the crystal lattice, which can introduce energy levels within the band gap. Fewer defects mean a more uniform and well-defined energy band structure, which can result in a larger band gap.

**Figure 3:**
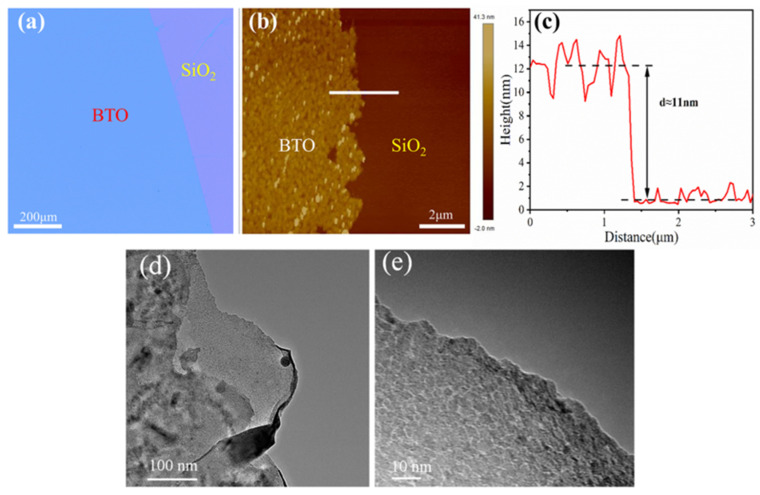
(**a**) Micrograph of annealed BTO film with an optical magnification of 20, (**b**) AFM image of BTO film, (**c**) variation of BTO thin film thickness along the BTO and SiO_2_ interface, (**d**,**e**) are the TEM images with a magnification of 5000× and 50,000×, respectively.

Figure 3 shows the morphology and thickness of the fabricated quasi-2D-BTO films. The morphology was observed at 20× magnification of a metallurgical microscope, where the colors of the SiO_2_ substrate and the 2D-BTO film are purple and blue, respectively (Figure 3a). It can be seen that good uniformity is obtained in the prepared BTO film, and an average thickness of ~11 nm was realized for the BTO film (Figure 3c). In TEM images (Figure 3d,e), there are some regions where the BTO film and substrate boundaries are closely connected and some regions where cracks are observed at the boundary between the BTO film and the substrate. The occurrence of these cracks can be ascribed to the differing thermal expansion coefficients of the thin films and substrates, resulting in varying rates of expansion and, consequently, inducing stress and cracking. In addition, BTO thin film exhibits greater roughness compared to the Si substrate. This phenomenon can be attributed to the annealing process, specifically the temperature and duration to which the film is subjected. During the annealing process, the BTO thin film may undergo various surface imperfections, such as wrinkling, cracking, or increased roughness due to the induced strain and stress.

**Figure 4:**
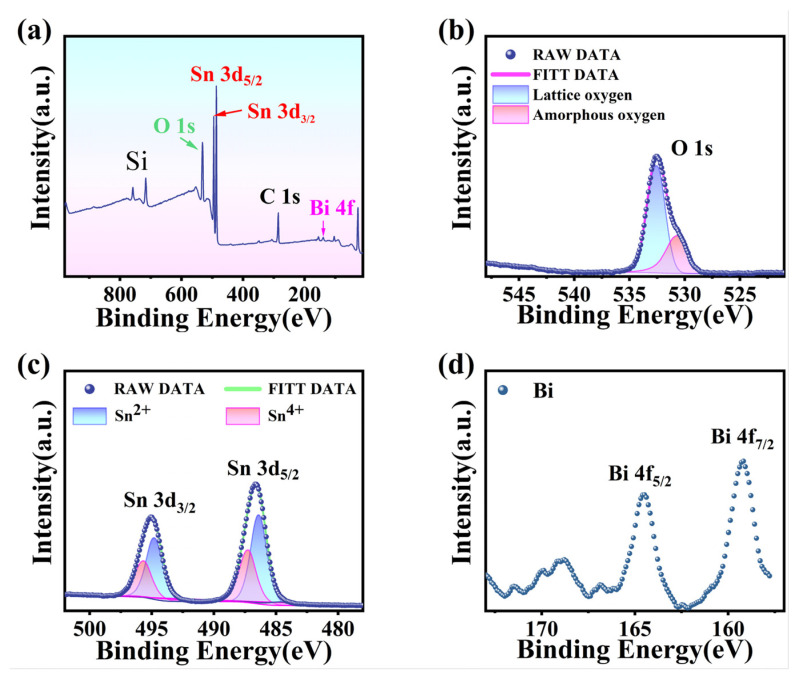
XPS spectra of quasi-2D-BTO thin film with (**a**) full spectrum, (**b**) O 1s fine spectrum, (**c**) Sn 3d fine spectrum, and (**d**) Bi 4f fine spectrum.

The X-ray photoelectron spectroscopy of the BTO films is presented in Figure 4. The element peaks of Sn, O, and Bi can be observed, and the atomic percentages of Sn, O, and Bi are 29.2%, 70.7%, and 0.1%, respectively. The Bi element was measured to be less than 0.2%, which can be interpreted as the Gibbs free energy (ΔG_f_) of BiO_2_ is lower than that of SnO_2_ [42]. According to the laws of thermodynamics, the metal oxide (SnO_2_) with the largest ΔG_f_ will cover the surface of the Bi-Sn alloy, and only a small amount of the metal oxide (BiO_2_) will be precipitated due to a lower ΔG_f_. The high-resolution XPS spectrum of O1s is fitted by the amorphous oxygen at 532.52 eV and the lattice oxygen at 530.58 eV. The amorphous lattice oxygen peak is much higher than that of lattice oxygen can be partially attributed to the large amount of oxygen adsorption on the surface of the 2D-BTO film. In addition, the amorphous lattice oxygen peak may partially come from the amorphous SiO_2_ substrate since its oxygen atoms are not arranged in regular, repeating crystal lattices. This lack of long-range order can lead to differences in the binding energy of the oxygen atoms and result in distinct peaks in the XPS spectrum. The peaks at 164.52 eV and 159.18 eV correspond to Bi 4f_5/2_ and Bi 4f_7/2_ of Bi_x_Sn_1−x_O_2_, respectively, which means that the bismuth element in the film is Bi^3+^. The Sn 3d_3/2_ and 3d_5/2_ peaks were located at 495.08 eV and 486.68 eV, respectively. The two Sn 3d peaks can be further fitted by the Sn^2+^ peak centered at 494.8 eV and 486.3 eV, and the Sn^4+^ peaks centered at 495.6 eV and 487.3 eV, respectively. This evidence further demonstrated the existence of both SnO and SnO_2_ in the resultant BTO films.

**Figure 5:**
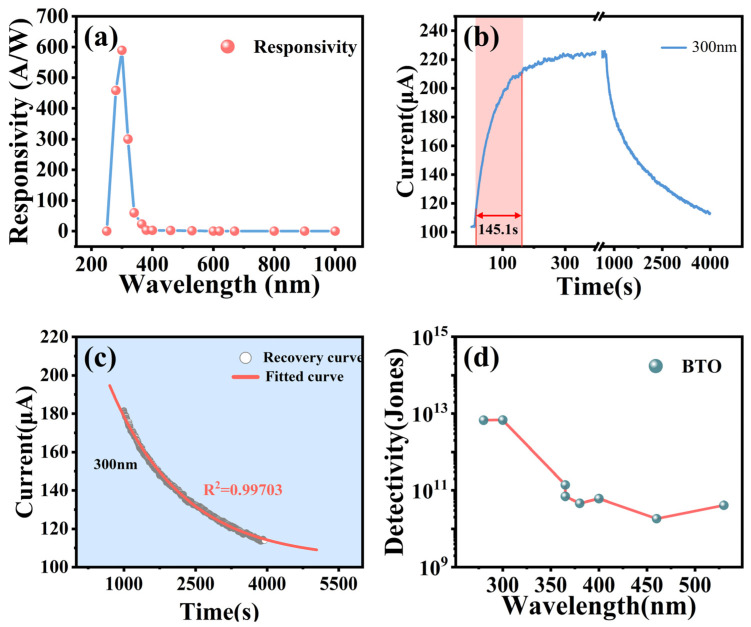
(**a**) Responsivity of annealed quasi-2D-BTO films to different wavelengths of light at 5 V bias, (**b**) photocurrent variation and response time of BTO films illuminated by 300 nm UV light with a power density of 45.5 μW/cm^2^, (**c**) recovery time function fitting of BTO films under 300 nm UV illumination, (**d**) detectivity (D*) of photodetectors based on BTO films.

The photocurrent was tested in the wavelength ranging from 200 nm to 1000 nm under a 5 V bias. The spectral responsivity (*R_λ_*) [43] of photodetectors is calculated by the following formula [44,45,46]:

(1) $$R_{\lambda }=\frac{I_{\mathrm{Ph}}-I_{\mathrm{Dark}}}{P_{\lambda }S}$$

where *R_λ_* is the responsivity, *I_Ph_* is the photocurrent, *I_Dark_* is the dark current, *P_λ_* is the density of the incident light, and *S* is the optical sensitive area. As depicted in Figure 5a, the responsivity of the BTO film increases rapidly as the wavelength increases from 250 nm to 300 nm, with a peak value of 589 A/W at 300 nm. Then, the responsivity reduces to a very low value during the wavelength range of 400–1000 nm, which is well consistent with the optical band gap of 3.10 eV. Thus, the 2D-BTO film can serve as highly sensitive visible-blind photodetectors. The high responsivity is attributed to the photocarriers trapped by the internal or surface defects/impurities in the quasi-2D-BTO with high surface area. From Figure 5a, it can be seen that the full width at half maximum (FWHM) of the responsivity for the film photodetector is 49 nm, which is much narrower than reported devices based on undoped SnO_2_ film and can be used as a narrowband UV photodetector.

The minimum detectable level of a photodetector can be described by the detection rate D* given by the following formula [39]:

(2) $$D^{*}=\frac{R_{\lambda }\sqrt{S}}{\sqrt{2qI_{dark}}}=\frac{R_{\lambda }}{\sqrt{2qJ_{dark}}}$$

where R_λ_ is the spectral responsivity, S is the effective area (S = 4.43 × 10^−3^ cm^2^), q is the electronic charge (*q* = 1.602 × 10^−19^ C), *I_dark_* is the dark current, and *J_dark_* is the dark current density. The calculated detection rate D* is shown in Figure 5d, from which it can be seen that in the 2D-BTO film photoconductor, the maximum value is about 6.82 × 10^12^ Jones.

Figure 5b shows the photocurrent generated under 300 nm light irradiation with a power density of 45.52 μW/cm^2^. The photocurrent initially increases dramatically and then gradually drops. The maximum photocurrent reaches ~224A. However, the response time is relatively slow, requiring 145.52 s (Figure 5b), which is not suitable for a good photoelectric detector. As shown in Figure 5c, the recovery speed is too slow, which presents a typical PPC effect. The photocurrent of the recovery process in 2D-BTO film can be fitted by the following equation [34]:

(3) $$I_{PPC}\left(t\right)=I_{PPC}(0)e^{-{\left(t/\tau \right)}^{\beta }}$$

where *τ* represents the lifetime of the photoinduced carriers, β is the decay exponent (0 < *β* < 1), and *I*_PPC_(0) is the buildup photocurrent at the moment of the light source being removed. The *β* = 0.69, τ = 1.65 × 10^3^ s for 2D-BTO films after 300 nm UV irradiation was calculated by fitting. The long recovery time can be attributed to the presence of internal or surface defects/impurities-related deep energy levels in the band gap of 2D-BTO films. The results of the 2D-BTO decay time fit after different solution treatments are listed in Table 1. It can be clearly seen that the solution treatment τ is significantly reduced. This indicates that the 2D-BTO film has a photocatalytic effect and the PPC effect can also be significantly reduced by this method. The lifetime of photo-induced carriers determines how long they are available to participate in chemical reactions [47]. A shorter carrier lifetime means the carriers recombine (electron and hole coming back together) more quickly. Rapid carrier recombination reduces the chances of these carriers reaching the material’s surface to participate in reactions. As a result, the photocatalytic efficiency is reduced. In addition, in BTO materials, the PPC effect is often related to the accumulation and trapping of charge carriers [48]. A reduced carrier lifetime means carriers recombine quickly instead of being trapped or accumulating in defect states. Thus, the material loses its ability to exhibit persistent photoconductivity.

The PPC effect and long recovery time of photoconductors are not beneficial for photodetector applications. The long recovery time is caused by the photocarriers trapping by the defects or impurities, and many unrecombined photocarriers still residue in the 2D-BTO after turning off the UV light. The recombination between photoelectrons and photoholes is retarded, and the reduction of photocurrent requires a long time. The rapid consumption of photocarriers, i.e., photoelectrons and photoholes, may assist in the recovery of photodetectors after turning off the UV light. It is well known that the photoholes have oxidative properties. Here, some reductive substances are employed to react with residual photoholes, and the recovery process of the photodetector is accelerated by the consumption of photocarriers through chemical reactions between photoholes and reductive substances.

**Figure 6:**
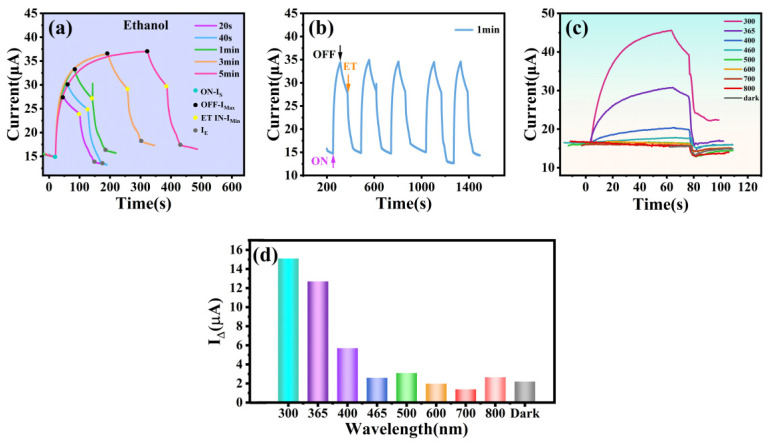
Response of BTO films to ethanol. (**a**) Current variation of BTO films under 365 nm UV light on/off and drops of ethanol added, the volume of ethanol is 5 μL. (**b**) Current variation of BTO films after dropwise addition of ethanol for 20s~5min after 365nm UV light illumination at applied voltage of 5 V. (**c**) Current variation of BTO films reacting with ethanol after irradiation at 300~800 nm light for 1 min. (**d**) The difference between the I_S_ and I_E_ of the prepared BTO films under irradiation with light of different wavelengths.

Figure 6 shows the photocurrent changes of the prepared 2D-BTO film with and without dripping of ethanol. Ethanol was added 1 min later after light off. In Figure 6a, the photocurrent was measured at different illumination times using 365 nm UV light at a temperature of 40 °C. ON-I_S_ represents the photocurrent without illumination, OFF-max shows the maximum photocurrent before the termination of the light source, ET IN-I_min_ represents the photocurrent when ethanol starts to drop, and *I_E_* is the final current. It can be seen that the photocurrent increases as the irradiation time increases and the maximum photocurrent reaches approximately 36.9 μA when the irradiation time reaches 5 min. After the light off, the photocurrent slowly decreases following a slow drop mode (I_Max_~I_s_). When introducing the ethanol steam to the photodetector, the photocurrent drops dramatically, and finally levels off. Herein, *I*_∆_ is introduced, which represents the difference between the *I_S_* and *I_E_*, as follows

(4) $$I_{\Delta }=I_{S}-I_{E}$$

Figure 6b shows the photocurrent variation of the 2D-BTO film under different irradiation wavelengths, ranging from 300 nm to 800 nm. It is found that the 2D-BTO film can only produce a large photocurrent in the wavelength range of 300 nm to 460 nm. Under the irradiation of light at wavelengths larger than 460 nm, the photocurrent can hardly be observed. Thus, the current variation in this wavelength range is essentially the same as that in the dark conditions. With the addition of ethanol, a rapid drop trend is observed. The obtained *I_∆_* values are $I_{\Delta 300nm}$ = 15.1 μA, $I_{\Delta 365nm}$ = 12.7 μA, $I_{\Delta 400nm}$ = 5.7 μA, and $I_{\Delta 460nm}$ = 2.6 μA, respectively (Figure 6d), which decrease with irradiation light wavelength increasing. Notably, the current will still decrease when ethanol is added in the dark, this is due to the fact that the test was performed under the condition of 5 V bias plus and the carriers generated by electrical excitation reacted with ethanol. Therefore, the photocatalytic response of the 2D-BTO film to ethanol should be the joint result of the action of both photoexcited carriers and electrically excited carriers [38]. After introducing the ethanol, the variation of dark current *I*_∆_ is 2.5 μA, which is much lower than the residual photocurrent after light off. Thus, the residual photocarriers mainly respond to the ethanol molecules. To check the reliability of the performance of the BTO film, a repeated experiment under 365 nm illumination was performed, as shown in Figure 6c. Similar behaviors of current curves are observed, which further demonstrate that the fabricated BTO film has excellent repetitive performance.

**Figure 7:**
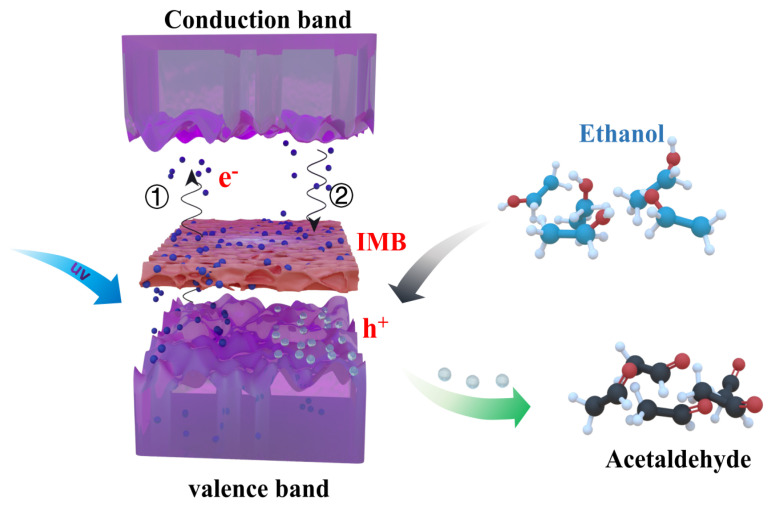
Schematic diagram of the electron transition process and the reaction mechanism with ethanol in BTO films.

Figure 7 illustrates the energy level and the reaction mechanism between the 2D-BTO film and ethanol. Under the excitation of UV light, the electrons in the 2D-BTO film are initially excited from the valence band to the conduction band (①). The existence of an intermediate band (IMB) originating from defects/impurities-related deep level can store photoelectrons from the conduction band otherwise directly recombine with the photoholes in the valence band, i.e., the photoelectrons are captured by the intermediate energy level (②), and then slowly jump to the valence band, resulting in a large number of photoholes residing in the valence band that cannot be consumed immediately. When the ethanol droplet contacts with the 2D-BTO, the “dark” photocatalysis process will take place, and the unrecombined photoholes will oxidize ethanol molecular to acetaldehyde, and the photoholes are consumed rapidly by oxidizing the ethanol to acetaldehyde with high reductive property [44]. Furthermore, the photoelectrons can be depleted by reacting with adsorbed O_2_ on the surface of the produced H^+^ from the photohole oxidation reaction. In addition, the photogenerated electrons separated in the conduction band diffuse to the surface of 2D-BTO can form a strong reduction potential with strong reducing properties to react with oxidizing substances such as CO_2_ to form CO, CH_4_, and other substances [49]. Photogenerated electrons can also reduce the oxygen on the surface of the polymer film to superoxide radical ·O_2_^−^. And ·O_2_^−^ undergoes a series of reactions to produce ·OH radicals, thus degrading organic matter [50].

**Figure 8:**
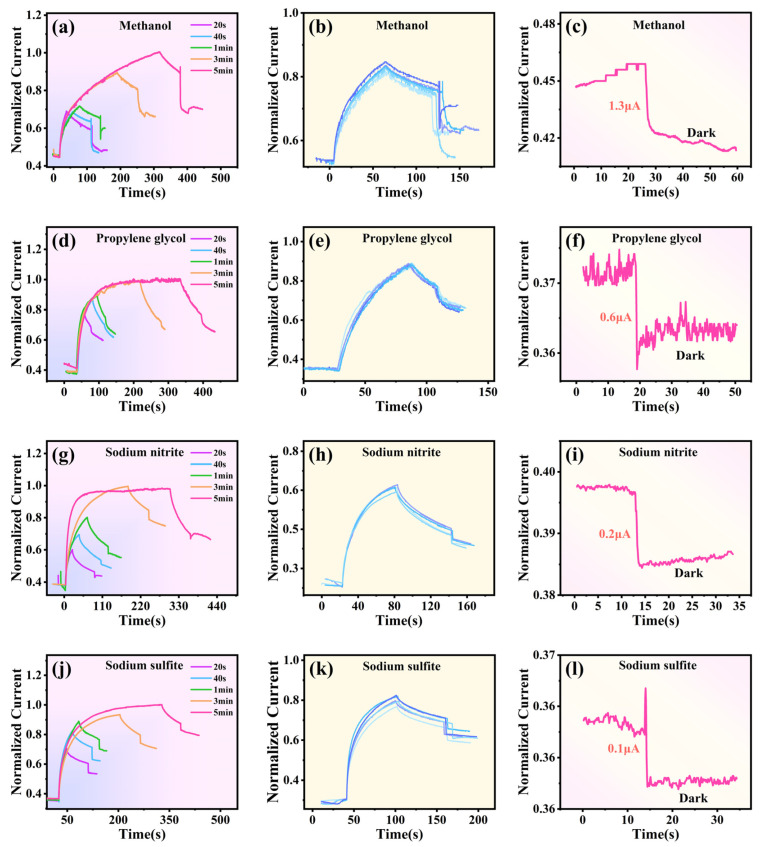
(**a**,**d**,**g**,**j**) Current changes of BTO thin film before and after adding methanol, propylene glycol, Na_2_SO_3_, and NaNO_2_ under different UV exposure times; (**b**,**e**,**h**,**k**) repeat current curves of dropping methanol, propylene glycol, Na_2_SO_3_, and NaNO_2_; (**c**,**f**,**i**,**l**) dark current changes of BTO thin film after adding methanol, propylene glycol, Na_2_SO_3_, and NaNO_2_.

Figure 8 reports the photocurrent variation of the 2D-BTO film before and after introducing methanol, propylene glycol, Na_2_SO_3_, and NaNO_2,_ respectively. The solutions of Na_2_SO_3_ and NaNO_2_ have concentrations of 0.1 mol/L each. The Na_2_SO_3_ solution was prepared by adding 12.6 g of Na_2_SO_3_ to 1L of deionized water and stirring until homogeneous. On the other hand, the NaNO_2_ solution was prepared by adding 5.3 g of Na_2_SO_3_ to 1L of deionized water and stirring until homogeneous. Next, the Na_2_SO_3_ and NaNO_2_ solutions were dropped in 1 min after the UV light off. Different UV light exposure times are explored, including 20 s, 40 s, 1 min, 3 min, and 5 min. After introducing the above substances, the decay of residual photocurrent changes from slow drop mode to rapid drop mode (Figure 8a,d,g,j). For methanol, the photocurrent initially decreased dramatically and then slowly decreased to the lowest value, but finally made a significant recovery. In contrast, the current in the samples dropped with propylene glycol always showed a downward trend. For the samples dropped with Na_2_SO_3_ and NaNO_2_, the current also shows a similar trend: it initially drops moderately, then decreases quickly, and finally shows a slow downward trend to the end.

The repeat experiments have been carried out (Figure 8b,e,h,k). Obviously, the sample dropped with propylene glycol shows the best reproducibility because the current variation almost maintains consistency. However, the reproducibility of the sample dropped with methanol is relatively poor, and the final current shows a large fluctuation. Figure 8c,f,i,l report the dark current change by adding these solutions without UV light illumination. A significant degree of the dark current drop is observed in the sample dropped with methanol, and the initial dark current was reduced by approximately 1.2 μA. Different degrees of the dark current drop were also observed in other cases. Notably, the dark current showed almost no change in the sample dropped with NaNO_2_, reducing only approximately 0.1 μA, which shows that NaNO_2_ is not a good candidate for UV photodetection and photocatalysis.

The photocurrent (I_Δ5min_) of 2D-BTO after 5 min of UV light irradiation and *τ* values after adding the solution are summarized in Table 1. The maximum I_Δ5min_ is obtained in the sample dropped with ethanol, realizing an average value of approximately 12.14 μA. In comparison, the minimum I_Δ5min_ value only has an average value of 1.22 μA, which is obtained in the sample dropped with NaNO_2_. Compared with other solutions, these results further suggested that ethanol is a promising candidate for UV photodetection and photocatalysis compared to other solutions.

By comparing the values of *τ* and *I_Δ_* in Table 1, it can be found that the sample without any chemical introduction shows a slow recovery speed with *τ* value of 1.65 × 10^3^ s. The recovery times of 2D-BTO greatly decrease after introducing the chemical solution. And by fitting Equation (2), it is calculated that the *τ* of the 2D-BTO after the introduction of ethanol reaches 5.71 s. It is noteworthy that the *τ* values after sodium sulfite and sodium nitrite treatment are very small only 2.53 × 10^−2^ s and 4.69 × 10^−2^ s, but I_Δ_ is also very small, indicating that these two solutions are well suited for application to devices with small photocurrents to eliminate the PPC benefit. A comparison of the dark current and recovery speed between the presented BTO thin films and the previous report for other micro/nanostructured SnO_2_ photodetectors was provided in Table 2. The results reported in this work are superior to those reported in reference [6,28,51], where the recovery time is greater than 10 s. Although references [4,5] exhibit a fast recovery time, the fabrication process of Sb-doped SnO_2_ nanowires is complicated and involves using many different techniques. In addition, the SnO_2_ microrod reported in reference [4] needs an accurate radius of curvature of the bent film and appropriate voltage, which still faces the problem of complexity and cost, while the photon responsivity in reference [38] is relatively small(i.e., 60A/W). In this case, the fabrication process is relatively simple and the used chemical liquids such as ethanol, methanol, and propylene glycol are easily available. The use of chemical liquids to eliminate PPC effects still deserves further exploration.

## 4. Conclusions

In this work, quasi-two-dimensional Bi_x_Sn_1−x_O_2_ (2D-BTO) thin films were prepared by a liquid metal transfer method. The fabricated 2D-BTO photodetectors exhibit a significant response to UV light, realizing the summit responsivity of 589 A/W at 300 nm and detectivity of 6.82 × 10^12^ Jones at 300 nm. After turning off the UV light, the photocurrent decreases slowly due to the PPC effect. By introducing ethanol, methanol, propylene glycol, Na_2_SO_3,_ and NaNO_2_ on the device surface, the photocurrent of 2D-BTO film rapidly reduces in a short time. And the recovery time of 2D-BTO film is reduced from 1.65 × 10^3^ s to 5.71 s. The rapid recovery can be attributed to the accelerated consumption of photocarriers by reacting with the introduced ethanol or other chemicals. This study demonstrates high-performance UV photodetectors based on quasi-two-dimensional oxides and provides an innovation route to reduce the recovery time of photoconductors with the PPC effect. Future work needs to be carried out to explore the long-term stability and the optimal concentration of the chemical liquids, as well as chemical solutions that can better eliminate PPC.

## Figures and Tables

**Table 1:** The 2D-BTO films with PPC fitting parameters for photodetectors after solution treatment.

Solution	τ(s)	β	I_Dark_	I_Δ5min_ (μA)
None	1.65 × 10^3^	6.98 × 10^−1^	15.6 μA	─
Ethanol (99.5%)	5.71	1.48 × 10^−1^	17.3 μA	12.2
Methanol (99.85%)	1.22 × 10^−1^	3.7 × 10^−2^	15.3 μA	6.2
Propylene glycol (99%)	9.41 × 10^−2^	5.44 × 10^−2^	20.3 μA	4.0
Sodium sulfite (99.9%)	2.53 × 10^−2^	4.79 × 10^−2^	12.1 μA	1.5
Sodium nitrite (99.5%)	4.69 × 10^−2^	5.40 × 10^−2^	11.8 μA	0.9

**Table 2:** Comparison of the dark current and recovery speed between the prepared BTO thin films and previous report for SnO_2_ photodetectors.

Photodetector	Dark Current	Responsivity	Recovery Time	Refs.
SnO_2_ monolayer nanofilm	60–90 μA/1 V	Not mentioned	>50 s	[28]
SnO_2_ nanowire array	77 µA/12 V	Not mentioned	>150 s	[51]
Sb-doped SnO_2_ nanowire	2 pA/1 V	6250 A/W	≈1	[5]
SnO_2_ nanowire	19.4 nA/1 V	Not mentioned	>50 s	[6]
SnO_2_ nanowire array	2 pA/1 V	Not mentioned	10 s	[52]
SnO_2_ microrod	13 µA/1 V	3 × 10^5^ A/W	<1 s	[4]
Bi_x_Sn_1−x_O_2_ (2D-BTO) (0.017 < x < 0.041)	0.25 nA	60 A/W	1 s	[38]
This work	15.6 µA/5 V	589 A/W	5.71 s	

## Data Availability

Data will be made available on request.
